# Construction of Hydrogen-Bonded Self-Assembled Structures of Sodium 4-[(4-Chlorobenzoyl) Amino] Benzoate for Dispersion and Lubrication in Polypropylene

**DOI:** 10.3390/molecules30030527

**Published:** 2025-01-24

**Authors:** Yapeng Dong, Fuhua Lin, Tianjiao Zhao, Meizhen Wang, Xinyu Hao, Dingyi Ning, Yanli Zhang, Kexin Zhang, Dan Zhou, Xiangyang Li, Jun Luo, Bo Wang

**Affiliations:** 1 School of Chemical Engineering and Technology, Taiyuan University of Science and Technology, Taiyuan 030024, China; s202221211035@stu.tyust.edu.cn (Y.D.); s202221211044@stu.tyust.edu.cn (T.Z.); s202321111073@stu.tyust.edu.cn (M.W.); s202421211097@stu.tyust.edu.cn (X.H.); s202421211098@stu.tyust.edu.cn (D.N.); 2021051@tyust.edu.cn (Y.Z.); 2022072@tyust.edu.cn (K.Z.); 2021053@tyust.edu.cn (D.Z.); 2 School of Traffic Engineering, Shanxi Vocational University of Engineering Science and Technology, Jinzhong 030619, China; linfuhua@sxgkd.edu.cn; 3 Department of Chemical and Chemical Engineering, Shanxi Polytechnic College, Taiyuan 030006, China; 4 Guangzhou Fibre Product Testing and Research Institute, Guangzhou 510220, China; luoj@iccas.ac.cn

**Keywords:** polypropylene, hydrogen-bonded self-assembled structure, dispersion, lubrication effect, crystallization behavior, rheological behavior

## Abstract

Among numerous nucleating agents, organic carboxylate nucleating agents have been demonstrated to markedly improve the crystallization of polypropylene (PP). However, poor dispersion in the PP matrix affects the modification effect. In this work, erucamide (ECM) and sodium 4-[(4-chlorobenzoyl) amino] benzoate (SCAB) form a hydrogen-bonded self-assembled structure to obtain the SCAB-ECM composite nucleating agent in order to improve the dispersion of SCAB in the PP matrix and to exert internal lubrication on the PP matrix. The molecular structure of the SCAB-ECM composite was investigated by using Fourier transform infrared spectroscopy (FT-IR), and the result showed that SCAB and ECM could form a hydrogen-bonded self-assembled structure after physical blending. The scanning electron microscopy (SEM) results visualized that ECM promoted the dispersion of SCAB due to the formation of hydrogen-bonded self-assembled structures by SCAB and ECM. The crystallization behavior was studied using differential scanning calorimetry (DSC). At the crystallization temperature of 135 °C, the K of PP, PP/ECM, PP/SCAB, and PP/SCAB-ECM were 0.0002, 0.0004, 1.1616, and 1.8539, respectively. The crystallization properties of PP/SCAB-ECM were the best, which was attributed to the fact that SCAB formed a hydrogen-bonded self-assembled structure with ECM, which promoted the dispersion of SCAB in the PP matrix. The results of the rheological behavior demonstrated that the ECM can act as a lubrication effect, which was also proved by flexural strength results.

## 1. Introduction

As one of the most widely used thermoplastic polymers, polypropylene (PP) has the advantages of excellent processability and chemical stability, which play an indispensable role in a variety of fields such as aerospace, automotive, and construction [1,2,3]. However, due to the poor crystallization properties of PP, the wider application of PP is limited [4]. To further improve the crystallization properties of PP, one of the most convenient and effective methods is to add nucleating agents which can significantly improve the crystallization properties of PP [5].

Currently, the PP nucleating agents that have been marketed include sorbitol, phosphate ester salts, amides, organic carboxylates, etc. [6,7,8]. However, the nucleating agent is usually a small molecule compound with poor dispersion in the PP matrix, which affects the modification effect [9,10]. Therefore, physical blending is usually used to improve the effect of the nucleating agents. At present, the commonly used blends mainly have stearate, inorganic particles, etc., which, through lubrication, isolation, and other physical effects, improve the dispersion of the nucleating agents [11,12].

Dong et al. [13] physically blended zinc stearate (Znst) with sodium 4-[(4-chlorobenzoyl) amino] benzoate (SCAB). The results showed that the crystallization temperatures of PP/SCAB and PP/SCAB-Znst were increased by 13.48 °C and 14.96 °C, respectively, compared with that of PP. The Znst could effectively promote the dispersion of the organic carboxylate nucleating agent in the PP matrix. At the same time, the long carbon chain structure of the Znst played the role of internal lubrication in the crystallization process of PP, which promoted the rearrangement of the molecular chain, and then better improved the crystallization performance of the PP. Zhang et al. [14] physically blended *N*,*N*′-dicyclohexylterephthalamide (TMB-5) with the nano-CaCO_3_. The results showed that the crystallinity of PP increased from 53.56% to 55.41% with the addition of the nano-CaCO_3_. The nano-CaCO_3_ part of the composite nucleating agent played an isolation role and prevented the agglomeration of the TMB-5, promoting the uniform dispersion of the TMB-5 in the PP matrix, which resulted in a better nucleation effect.

Although the method of physical blending is relatively simple, the blends and nucleating agents are combined through chemical interactions such as hydrogen bonding to form a self-assembled structure, which theoretically can obtain a better dispersion effect, and has become a highlight in current research [15].

Ding et al. [16] found that the hydroxyl group on Tween-60 formed hydrogen bonds with the amino group on TMB-5, which broke the hydrogen bonds between TMB-5 that could lead to the agglomeration of the TMB-5, thus improving the dispersion and solution of the TMB-5, which led to the formation of self-assembled structures with efficient nucleation ability. The results showed that the nucleation efficiency of the TMB-5 was higher in the presence of Tween-60, which could increase the β-crystal content of PP from 44.7% to 71.6%. Roy et al. [17] found that the silanol group of polyhedral oligomeric silsesquioxane (POSS) intermolecularly bonded with the hydroxyl group of di(benzylidene) sorbitol (DBS) to form a hydrogen-bonded self-assembled structure, which led to the improvement of the dispersion of the nucleating agent, and then improved the nucleation efficiency. The results showed that the viscosity of the nucleating agent was about three orders of magnitude lower than that of the PP melt after the addition of POSS-DBS, and the rheological behavior study verified that the dispersion of the nucleating agent was significantly improved. Wang et al. [18] investigated the hydrogen bonding between the hydroxyl group on glass fiber (GF) and the amide group of TMB-5, which could enable the TMB-5 to form a self-assembled structure for efficient nucleation according to a regular arrangement on the surface of the GF to promote the dispersion of the nucleating agent, thus improving the nucleation efficiency of the TMB-5.

**Figure 1:**
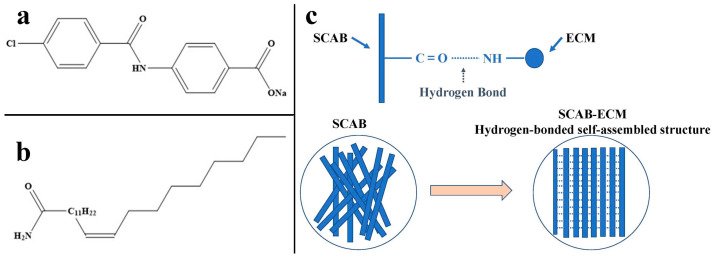
(**a**) The molecular structure of SCAB; (**b**) the molecular structure of ECM; (**c**) the mechanism of hydrogen bonding self-assembly structure formation between SCAB and ECM.

Among the numerous nucleating agents, the organic carboxylate nucleating agents have been demonstrated to markedly improve the crystallization of PP [16]. Most importantly, the low processing odor is particularly suitable for industry applications. SCAB (Figure 1a) is an exclusive organic carboxylate nucleating agent for polyethylene [19,20]. The microscopic morphology of SCAB is a slender strip, which has great potential to be applied in the process of PP modification, which can provide favorable conditions for the crystallization of PP. Compared with PP, the polarity of SCAB is more pronounced, resulting in poor dispersion. Constructing self-assembled structures with hydrogen bonding is an important way to improve the effect of SCAB. Erucamide (ECM, Figure 1b) is a common slipping agent used in the processing of plastics, which contains an amide group in the chemical structure of ECM [21,22,23]. The electronegativity differential between the nitrogen and carbonyl carbon atoms in the amide group gives rise to an unequal distribution of the electron cloud. Secondly, ECM may be able to form intermolecular interactions such as hydrogen bonding or Van Der Waals force with polar molecules [24]. Consequently, following the physical blending of SCAB with ECM, the amide groups between them may form a hydrogen-bonded self-assembled structure (Figure 1c), thereby promoting the uniform dispersion of SCAB in the PP matrix and improving the effect. In addition, the long carbon chain structure of ECM may act as an internal lubricant in the PP matrix, which evenly disperses inside the PP matrix and reduces the mutual friction between the PP molecular chains, thus further improving the effect of SCAB/ECM.

In this study, to further investigate the effect of hydrogen-bonded self-assembled structure on the dispersion of SCAB in the PP matrix, SCAB and ECM were physically blended to obtain the composite nucleating agent SCAB-ECM, and the molecular structure of SCAB-ECM was investigated by using Fourier transform infrared spectroscopy (FT-IR). The microstructure of the SCAB-ECM composite nucleating agent was observed by scanning electron microscopy (SEM). The PP/SCAB-ECM composites were prepared by melt blending, and the crystallization properties of the PP/SCAB-ECM composite were investigated by differential scanning calorimetry (DSC), the isothermal crystallization kinetics of the PP/SCAB-ECM composite was investigated by using Avrami equation, and the isothermal crystallization thermodynamics of the PP/SCAB-ECM composite was investigated by using Arrhenius’s equation and Laurizen–Hoffmann’s equation. The crystal structure of the PP/SCAB-ECM composite was observed using a polarizing microscope (POM). The rheological properties of the PP/SCAB-ECM composite were investigated using a rotational rheometer to describe the lubricating effect of the composite nucleating agent. Finally, the mechanical properties of the PP/SCAB-ECM composite were investigated.

## 2. Result

### 2.1. FT-IR Spectra of the SCAB-ECM Composite Nucleating Agent

**Figure 2:**
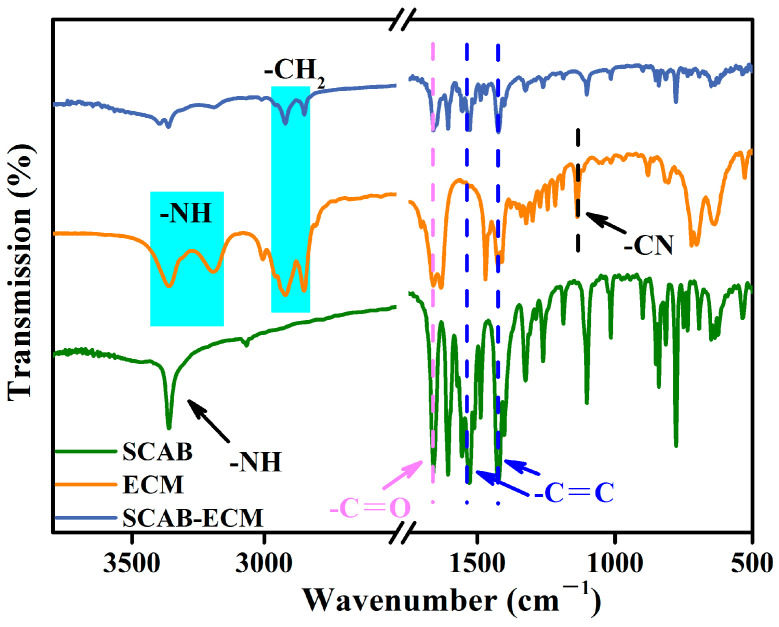
The FT-IR spectra of the SCAB-ECM composite nucleating agent.

The FT-IR spectra of the SCAB-ECM composite nucleating agent are shown in Figure 2. The characteristic absorption peak at 3367 cm^−1^ was -NH in the SCAB, the vibration peak at 1605 cm^−1^ represented the C=O, and the absorption peaks at 1525 cm^−1^ and 1417 cm^−1^ represented the C=C double bond of the benzene ring in the SCAB. The characteristic peaks of the ECM appeared at 3362 cm^−1^ and 3194 cm^−1^ which were the stretching vibrations of the -NH. The double peaks at 2921~2851 cm^−1^ were the characteristic peaks of -CH2 stretching vibration. The characteristic peak range of C=O stretching vibration was at 1661~1633 cm^−1^. The absorption peak at 1135 cm^−1^ represented -CN. In the ECM molecule, the amide group contained a carbonyl group (-C=O) for which stretching vibration forms a strong peak. After the SCAB and ECM were physically blended, the new characteristic peak corresponding to -CH2 appeared in the SCAB, which proved that the physical blending was successful. However, the characteristic peak -NH of the ECM moved to the left, which formed two weak peaks. The characteristic peak -C=O of the SCAB-ECM composite weakened compared with the SCAB. The results indicated that the stable self-assembled structure formed by hydrogen bonding interactions between the amide groups of ECM and SCAB may facilitate the dispersion of SCAB in PP, which improved the nucleation efficiency [25].

### 2.2. SEM Photograph of the SCAB-ECM Composite Nucleating Agent

**Figure 3:**
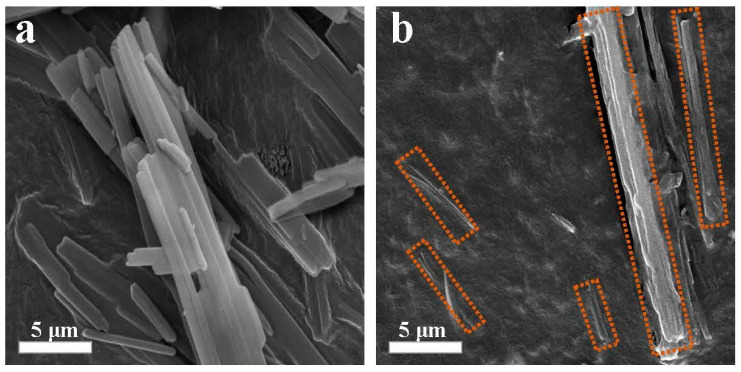
The SEM photograph of the SCAB-ECM composite nucleating agent. (**a**) SCAB; (**b**) SCAB-ECM.

The microscopic morphology of SCAB and SCAB-ECM was observed with SEM, and the results are shown in Figure 3. As illustrated in Figure 3a, the microscopic morphology of the SCAB is characterized by smooth, slender strips that are closely arranged. This leads to agglomeration in the PP matrix, thus affecting the modification effect. As shown in Figure 3b, the dash lines were labeled the SCAB-ECM and it can be seen that the ECM was wrapped around the surface of the slender strips due to the formation of hydrogen-bonded self-assembly structure between the SCAB and ECM, and the slender strips showed a tendency to separate, which indicated that the dispersion of SCAB in the PP matrix can be improved, thus improving the modification effect.

### 2.3. Non-Isothermal Crystallization Behavior of the PP/SCAB-ECM Composite

**Figure 4:**
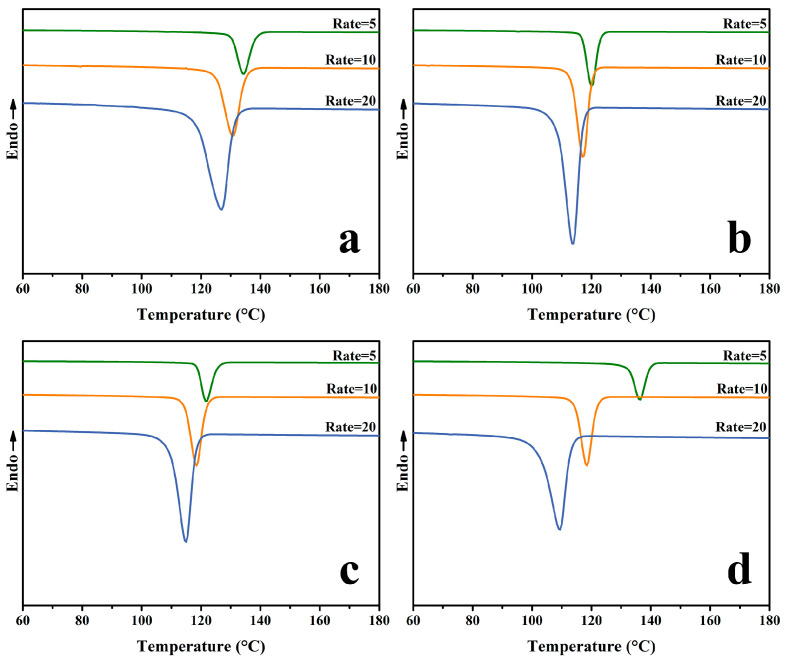
Non-isothermal DSC curves of the samples at different cooling rates. (**a**) PP; (**b**) PP/SCAB; (**c**) PP/ECM; (**d**) PP/SCAB-ECM.

The non-isothermal crystallization curves of the samples are given in Figure 4 at 5 °C/min, 10 °C/min, and 20 °C/min, respectively. As the cooling rate increased, the crystallization peaks of the PP/SCAB-ECM composite widened gradually and the crystallization temperature (*T*_c_) moved towards a low temperature. The result from the rate of the PP molecular chain folding into the crystallization lattice lags behind the rate of decreased PP temperature at the high cooling rate, resulting in prolonged crystallization time, and the extended crystallization time moves to the low-temperature direction by the peak of the crystallization temperature [26].

### 2.4. Isothermal Crystallization Kinetic Analysis of the PP/SCAB-ECM Composite

**Figure 5:**
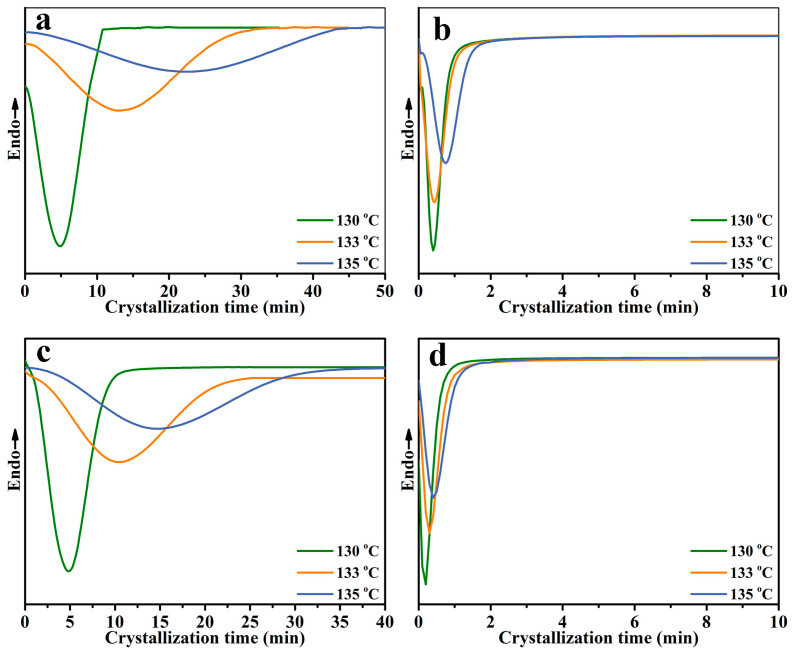
Isothermal crystallization curves of the samples at different temperatures. (**a**) PP; (**b**) PP/SCAB; (**c**) PP/ECM; (**d**) PP/SCAB-ECM.

The isothermal crystallization curve of the samples at 130 °C, 133 °C, and 135 °C is shown in Figure 5. It can be visualized from the figure that the different samples have a single crystallization peak at each crystallization temperature. From the width of the crystallization peaks, it can be seen that the crystallization time became longer as the crystallization temperature increased. Meanwhile, the crystallization peak width of the PP/SCAB-ECM composite at the same crystallization temperature was PP/SCAB-ECM < PP/SCAB < PP < PP/ECM. The results showed that the addition of the SCAB accelerated the crystallization rate of the PP, and the dispersion of the self-assembled structure formed by the ECM and SCAB with hydrogen bonding in the PP matrix was improved, which further accelerated the crystallization rate.

The isothermal crystallization kinetics of the samples were compared with that of the samples. On the basis of a linear relationship between the evolution of crystallinity and the evolution of heat released in the crystallization process, the relative crystallinity X_w_(t) was calculated by integrating the exothermic peak with Equation (1).

(1) $$X_{w}\left(t\right)=\int_{t_{0}}^{t}(\frac{\mathrm{dH}}{\mathrm{dt}})\mathrm{dt}/\int_{t_{0}}^{t_{\infty }}(\frac{\mathrm{dH}}{\mathrm{dt}})\mathrm{dt}$$

**Figure 6:**
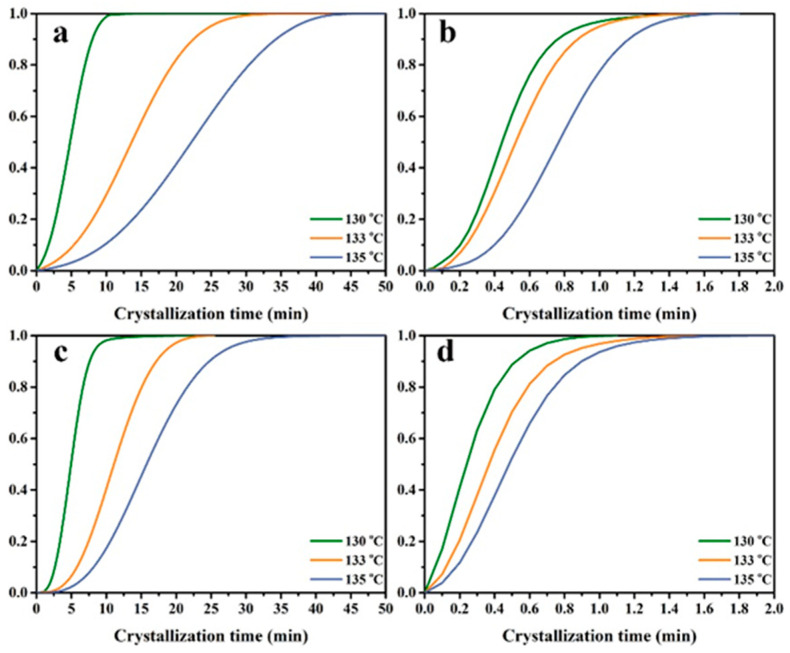
Relative degree of crystallinity with the time of the samples. (**a**) PP; (**b**) PP/SCAB; (**c**) PP/ECM; (**d**) PP/SCAB-ECM.

In Equation (1), dH/dt is the crystallization heat flow rate at a temperature of t. t_0_ and t_∞_ denotes the temperature at the beginning of crystallization and the temperature at the completion of crystallization, respectively. Through Equation (1), X_w_(t) at any crystallization temperature can be converted to the volume fraction relative crystallinity X_v_(t), which in turn yields the relative crystallinity of the PP/SCAB-ECM composite at different isothermal crystallization temperatures with the crystallization time change curve shown in Figure 6.

As one of the important parameters describing the crystallization kinetics of the polymer, the half-time of crystallization can be read directly from Figure 5 which is denoted as t_1/2_ which is recorded in Table 1. The Avrami equation during the isothermal process is shown in Equation (2):

(2) log[-ln(1 -Xv(t))]=nlogt+logK

**Figure 7:**
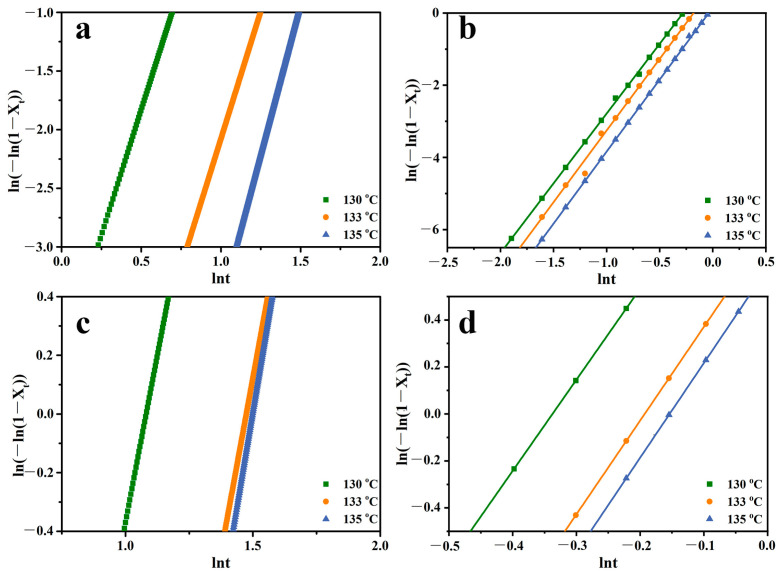
Isothermal crystallization kinetics curves of the samples. (**a**) PP; (**b**) PP/SCAB; (**c**) PP/ECM; (**d**) PP/SCAB-ECM.

Assuming a constant growth rate of polymer spherocrystals, *log*[−*ln*(1 − *X_v_(t)*)] − *logt* was plotted according to Equation (2) for which the slope of the line is the Avrami exponent n, and the intercept is the crystallization rate constant of the *logK* of the sample. The isothermal crystallization kinetics of the samples analyzed by the Avrami equation is shown in Figure 7. The data were recorded in Table 1. It was easy to see from the figure that the kinetic curves of the PP/SCAB-ECM composite showed a linear relationship.

It can be seen from Table 1 that the samples exhibited enhanced crystallinity (*X_c_*) at a crystallization temperature of 133 °C. The addition of the SCAB resulted in a notable improvement in the crystallinity of the PP which provided evidence that SCAB has the capacity to facilitate the crystallization properties of PP. The dispersion of the SCAB within the PP matrix was enhanced after the physical blending of the SCAB and ECM which further improved the *X_c_* of the PP. The mean value of n for pure PP at three isothermal crystallization temperatures was 4.58, which indicated that the PP grew in the mode of homogeneous nucleation. The mean value of n for PP/ECM was 4.82, which indicated that the addition of ECM did not alter the nucleation mode of the PP. The mean value of n for PP/SCAB and PP/SCAB-ECM at three isothermal crystallization temperatures were 3.93 and 3.96, respectively. The results demonstrated a shift in the mode of growth for the PP, which exhibited a tendency towards a coexistence of homogeneous and heterogeneous nucleation [27,28].

At the crystallization temperature of 135 °C, the *K* of PP, PP/ECM, PP/SCAB, and PP/SCAB-ECM were 0.0002, 0.0004, 1.1616, and 1.8539 min^−n^, respectively. The result showed that the order of crystallization rate from fast to slow was PP/SCAB-ECM, PP/SCAB, PP/ECM, PP. The results demonstrated that the addition of ECM played a minor effect on the crystallization properties of PP, and increased the crystallization rate of PP slightly. After the addition of the SCAB, the SCAB played the role of heterogeneous nucleation in the PP matrix, which dramatically increased the crystallization rate of the PP and improved the crystallization properties of the PP. The addition of SCAB-ECM further increased the crystallization rate as well as the crystallinity. As a result, the formation of hydrogen-bonded self-assembled structure promoted the dispersion of SCAB-ECM uniformly in the PP matrix, provided more nucleation sites, and led to the regular arrangement of the PP molecular chains, which significantly improved the crystallization properties of PP [29,30,31]. The results of *X_c_* and t_1/2_ also verified the conclusion.

### 2.5. Isothermal Crystallization Thermodynamic Analysis of the PP/SCAB-ECM Composite

The crystallization activation energy ΔE, nucleation parameter *K_g_*, and surface free energy σ_e_ at the ends of the molecular chains of the PP/SCAB-ECM composite were calculated by introducing Arrhenius and Laurizen–Hoffmann equations in order to study the crystallization growth ability.

The ΔE of the polymer can be calculated using the Arrhenius equation, as demonstrated in Equation (3) [32]:

(3) $$K^{1/n}=k_{0}\exp(-\frac{\Delta E}{RT_{c}})$$

The logarithm of both sides of Equation (3) yields Equation (4):

(4) $$\left(1/n)\mathrm{lnK}=\ln k_{0}\;-\;\Delta E/(RT_{c}\right)$$

**Figure 8:**
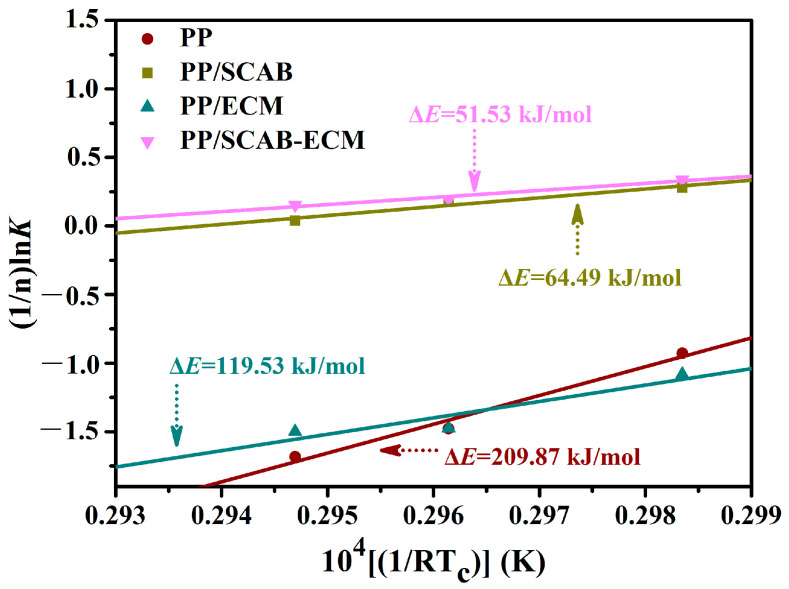
(1/n)lnK versus 1/(RTc) for the PP/SCAB-ECM composite.

In Equation (4), k_0_ represents a temperature-dependent pre-finger coefficient, R denotes the gas constant (8.314 J/ (mol K)), and *K* signifies the crystallization rate of the polymer. The slope was plotted against 1/(RT_c_) using (1/n)lnK, and a linear fit yielded a slope of ΔE as in Figure 8, and the resulting data were recorded in Table 2.

The Avrami equation describes the overall crystal behavior of polymers, including nucleation and growth. The Laurizen–Hoffmann equation provides a description of the nucleation process of spherical crystals [33].

(5) $$G=G_{0}\exp(-\frac{U^{*}}{R(T_{c}-T_{\infty })})\exp(-\frac{K_{g}}{T_{c}\times \Delta T\times f})$$

where G_0_ refers to the pre-factor, whereas U* represents the migration activation energy, which is 6.28 KJ/mol. f = 2T_c_/((T^0^_m_ + *T_c_*)) is the melting heat correction temperature. The degree of undercooling is given by ΔT = T^0^_m_ − *T_c_*. T_∞_ = T_g_ − C is expressed as the characteristic temperature of the polymer. The C and U* values of 60 K and 6280 J/mol, respectively, can be applied to the majority of polymers.

By taking logarithms on both sides of Equation (5) and defining lnG_0_ = a, we obtained Equation (6):

(6) $$\mathrm{lnG}+\frac{U^{*}}{R(T_{c}-T_{\infty })}=a-\frac{1}{T_{c}\Delta T_{f}}K_{g}$$

**Figure 9:**
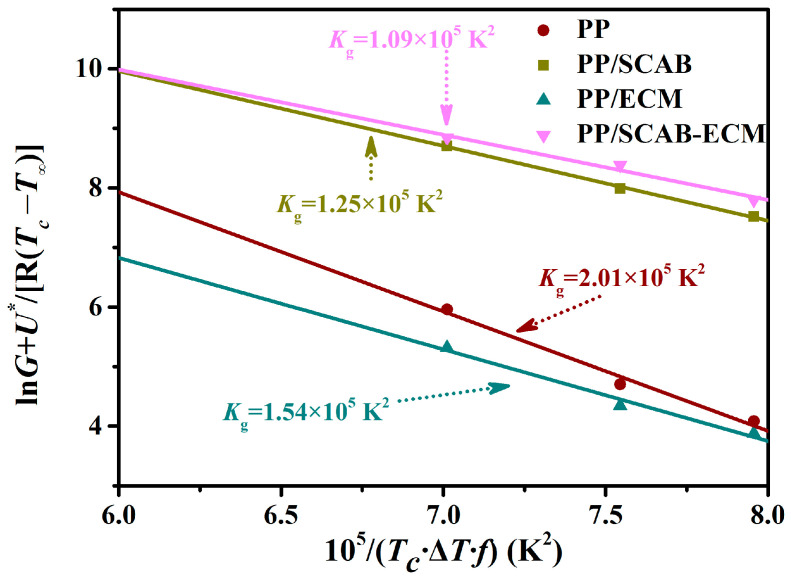
lnG + $\frac{U^{*}}{R(T_{c}-T_{\infty })}$ versus $\frac{1}{T_{c}\Delta T_{f}}$ for the PP/SCAB-ECM composite.

According to Equation (6), we plotted $\;lnG+\frac{U^{*}}{R(T_{c}-T_{\infty })}$ against $\frac{1}{T_{c}\Delta T_{f}}$. The linearly fitted slope was K_g_ and the value of σ_e_ was subsequently calculated using *K_g_*. The data were recorded in Figure 9 and Table 2.

K_g_ is the nucleation parameter which varies with different nucleation methods and Equation (7) is as follows [34]:

(7) $$K_{g}=4b_{0}\sigma \sigma_{e}T_{m}^{0}/\Delta H_{f}K$$

where σ is the surface free energy per unit area of the folded chain side and σ_e_ is the surface free energy per unit area of the molecular chain end; b_0_ is the thickness of the monomolecular layer, which is determined by the cellular parameters; *ΔH_f_* is the theoretical enthalpy of melting of the polymer; and K is the Boltzmann’s constant, *K* = 1.38065 × 10^−23^ J/K.

σ can be found from the equation σ = ab_0_ΔH, where a is an empirical constant of 0.1; for PP, b_0_ = 6.26 × 10^−10^ m; ΔH = 1.96 × 108 J/m^3^. Therefore, the free energy of the side surface of the folded chain of PP, σ = 130.83 erg/cm^2^, which was brought into Equation (7), and σ_e_ was obtained by calculating it from the known value of *K_g_*, and the data were recorded in Table 2.

It can be observed in Table 2 that the addition of the SCAB-ECM composite nucleating agent to the PP resulted in a reduction in *ΔE*, *K_g_*, and σ_e_. The result suggests that the SCAB-ECM composite nucleating agent reduced the activation energy required for PP crystallization, which increased the nucleation rate of PP. Furthermore, the SCAB-ECM composite nucleating agent reduced the interfacial free energy perpendicular to the molecular chain direction, which facilitated the folding and growth of the molecular chains on the nucleus, thus promoting the improvement of PP crystallization properties. Compared to the PP, the isothermal crystallization rate *K* of PP/ECM increased slightly and *K_g_* decreased substantially to about half of that of the PP, while the magnitude of *K_g_* was mainly determined by the nucleation rate rather than the crystallization rate. The result indicated that the ECM played a more important role in facilitating the PP nucleation process.

In addition, the *ΔE*, *K_g_*, and σ_e_ of the PP/SCAB-ECM composite were minimum. The result proved that the addition of SCAB-ECM could significantly improve the crystallization properties of PP, which was mainly due to the fact that hydrogen bonding between the SCAB and ECM amide groups resulted in the formation of self-assembled structure, which enabled the uniform dispersion of the SCAB-ECM composite within the PP matrix. The heterogeneous nucleation process was improved, and the crystallization properties of PP were improved.

### 2.6. POM of the PP/SCAB-ECM Composite

**Figure 10:**
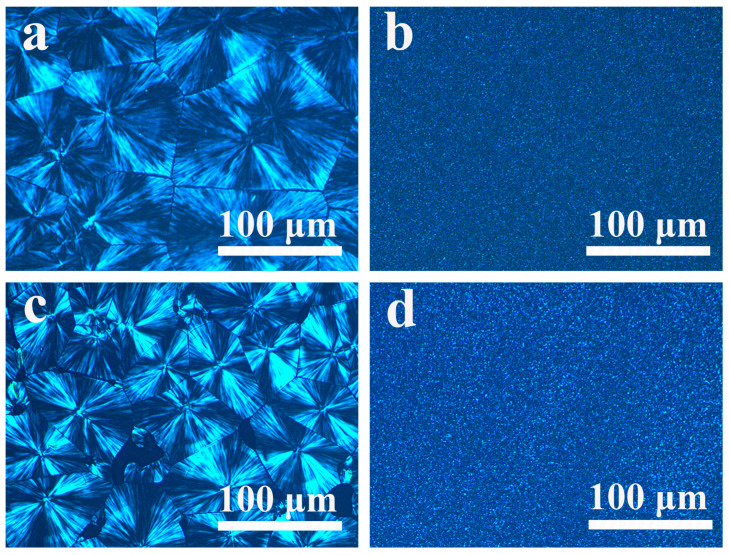
The POM morphology of the samples at 135 °C. (**a**) PP; (**b**) PP/SCAB; (**c**) PP/ECM; (**d**) PP/SCAB-ECM.

It can be clearly seen from Figure 10 that the spherical crystals of pure PP and PP/ECM were larger than 100 μm, which showed an obvious black cross-extinction phenomenon, clear boundaries of the spherical crystals, and a small number of crystal nuclei [35]. At the same scale of observation, the addition of SCAB and SCAB-ECM resulted in a point-like distribution of PP crystals, significant refinement of spherulites, and a sharply increased number of crystal nuclei. Figure 10b,d cannot visually compare the grain size. However, the results of the crystallization behavior study verified that the spherical crystal size of PP/SCAB-ECM was smaller under the same conditions as a result of the formation of the hydrogen-bonded self-assembled structure of SCAB and ECM in the PP matrix, which allowed for better dispersion and smaller spherical crystal size.

### 2.7. Lubrication Mechanism of the PP/SCAB-ECM Composite

**Figure 11:**
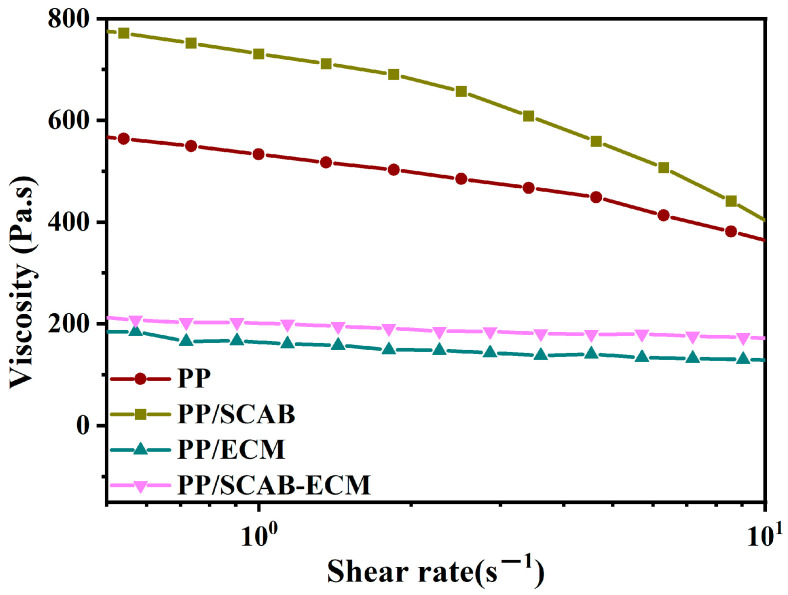
Relationship between viscosity and shear rate of the PP/SCAB-ECM composite at 200 °C.

The relationship between the viscosity and shear rate of the PP/SCAB-ECM composite at 200 °C is shown in Figure 11. It can be observed that under identical shear rates, the viscosity of the samples was as follows: PP/SCAB > PP > PP/SCAB-ECM > PP/ECM. The viscosity of PP/SCAB was higher than that of PP. This may be due to the aggregation of SCAB in the PP matrix. The phenomenon demonstrated that ECM can facilitate the rearrangement of PP molecular chains in the shear direction. Furthermore, the ECM exhibited superior internal lubrication effects on PP.

The rheological properties of the PP/SCAB-ECM composite were required for further investigation by the Arrhenius equation [36]:

(8) $$\eta^{*}=A\exp(\Delta E/RT)$$

where η* is viscosity, ΔE is viscous flow activation energy, *A* is viscosity constant, R is gas constant (8.314 J/mol∙K), and T is shear temperature.

**Figure 12:**
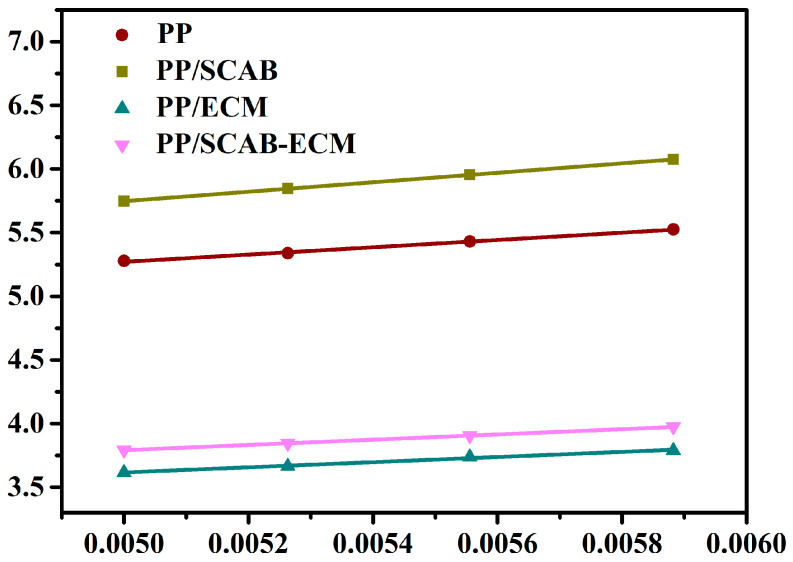
Relationship between the viscosity and temperature of the PP/SCAB-ECM composite.

According to Equation (8), we plotted lnη* against 1/T, with a slope of *ΔE*/R and an intercept of lnA. It can be seen from Figure 12 and Table 3 that lnη* shows a clear linear relationship with 1/T, indicating that the melt flow of the PP/SCAB-ECM composite fully conformed to the Arrhenius equation. The order of *lnA* and *ΔE* for the samples was as follows: PP/SCAB > PP > PP/SCAB-ECM > PP-ECM. The result indicated that ECM exhibited internal lubrication in PP, which could effectively reduce the interaction force between the PP molecular chains. The flexibility of the PP molecular chain was improved and the crystallization ability of PP was greatly increased. [37,38]. Meanwhile, SCAB-ECM could be better dispersed in the PP matrix due to the formation of hydrogen-bonded self-assembled structures.

### 2.8. Mechanical Properties of the PP/SCAB-ECM Composite

**Figure 13:**
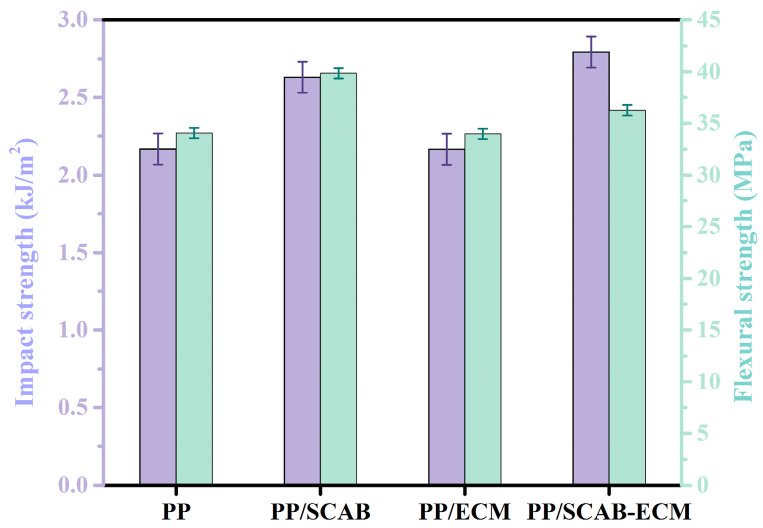
Mechanical properties of the PP/SCAB-ECM composite.

The mechanical properties of the PP/SCAB-ECM composite are shown in Figure 13. It can be seen from the figure that the addition of ECM had no effect on the impact strength of PP. The impact strength of PP/SCAB and PP/SCAB-ECM was increased by 21.8% and 30.1%, respectively, compared with PP. The addition of SCAB facilitated the refinement of grain size, the improvement of crystal structure, and the augmentation of interfacial bonding strength between spherical crystals, which was supported by the results of the POM. These improvements collectively improved the impact strength of the PP. The addition of the blend ECM could promote the dispersion of SCAB in the PP matrix, which exerted better the role of SCAB heterogeneous nucleation. Concurrently, the hydrogen-bonded self-assembled structure was formed by the SCAB and ECM that was more evenly dispersed in the PP matrix and more effectively exerted the nucleation effect, thereby further improving the impact strength of the PP.

In addition, the flexural strength of PP/SCAB increased by 5.8 MPa compared to the PP, which was attributed to the increase in X_c_, which accelerated the molecular chains to be rearranged tightly. The flexural strength of PP/ECM was slightly reduced. The flexural strength of PP/SCAB-ECM was only increased by 2.9 MPa. Although the *X_c_* of PP/ECM and PP/SCAB-ECM were increased, the ECM played the role of internal lubrication in the PP matrix to reduce the PP intermolecular forces, and when it was deformed by external forces, the molecular chains could slip and rotate with each other, which made the internal friction between the PP molecules decrease, resulting in the decrease in the flexural strength [39]. The impact strength of the PP was the highest with the addition of SCAB-ECM. The flexural strength of PP/SCAB-ECM was slightly improved compared to the PP. However, the crystallization properties of the PP were significantly improved. The result indicated that the modification effect on PP is beneficial.

## 3. Materials and Methods

### 3.1. Materials

PP (045) with a melt flow index of 3.5 g/10 min and polymer dispersity index of 5.4 was supplied by Shandong Orient Hongye Chemical Co., Ltd. (Shouguang, China). SCAB was purchased from Shanxi Advance Science Green Industry Research Institute Co., Ltd. (Xi’an, China). ECM was purchased by Shanghai Macklin Biochemical Technology Co., Ltd. (Shanghai, China).

### 3.2. Preparation of the SCAB-ECM Composite Nucleating Agent

The SCAB and ECM with a mass ratio of 1:1 (*w*/*w*) were mixed in a high-speed mixer (SHR10L, Zhangjiagang Jainuo Machinery Co., Ltd., Suzhou, China) for 2 min to obtain the SCAB-ECM composite.

### 3.3. Characterization of SCAB-ECM Composite Nucleating Agent

The FTIR spectrometer (Nicolet iS10, Thermo Scientific Inc., Waltham, MA, USA) was used to characterize the molecular structure of the SCAB-ECM composite nucleating agent through 64 scans per sample.

The microstructure of the SCAB-ECM composite nucleating agent was characterized by the SEM (Quanta 250 FEG, FEI, Hillsboro, OR, USA) at an accelerated voltage of 10 kV.

### 3.4. Preparation of the PP/SCAB-ECM Composite

The PP/SCAB-ECM composite formulation is presented in Table 4. The PP/SCAB-ECM composites were mixed in a high-speed mixer. Evenly mixed samples were fed into a micro-twin screw extruder. (WLG10A, Shanghai Xinshuo Precision Machinery Co., Ltd., Shanghai, China) for extrusion to obtain the PP/SCAB-ECM composite, which was molded for standard test specimens by a microinjection machine (WZS10D, Shanghai Xinshuo Precision Machinery Co., Ltd., Shanghai, China) with an injection pressure of 0.2 MPa at 200 °C.

### 3.5. Characterization of the PP/SCAB-ECM Composite

The non-isothermal and isothermal crystallization behavior of the PP/SCAB-ECM composite in a nitrogen atmosphere (50 mL/min) was studied using DSC (Q1000, TA company, Boston, MA, USA). Non-isothermal crystallization process: The sample was cooled from 220 °C to 30 °C at different cooling rates (5, 10, 20 °C/min). Then, it was heated to 220 °C at a rate of 10 °C/min. The crystallization process was recorded during these steps. Isothermal crystallization process: The sample was heated from 40 °C to 220 °C at a rate of 10 °C/min to eliminate its thermal history, and then held at the temperature for 3 min. Subsequently, the sample was cooled to 130 °C, 133 °C, and 135 °C at a rate of 10 °C/min for 30 min at each temperature. The isothermal curve was recorded for each cooling step.

The POM observation of the PP/SCAB-ECM composite was performed with a polarized optical microscope (DM2700, Leica, Wetzlar, Germany) and combined with a hot stage (THMS 600, Linkam, Surrey, UK) to control the temperature for 30 min. at 135 °C.

The rheological properties of the PP/SCAB-ECM composite were evaluated using a rotational rheometer (DHR-2, TA Company, Boston, MA, USA) in the oscillating mode. The test procedure was performed in the dynamic mode and 25 mm parallel plate geometry with a gap setting of about 2 mm. The test temperatures were set at 180 °C, 190 °C, 200 °C, and 210 °C, and the scanning frequency was settled at 0.1 Hz.

The Izod impact test machine (TY-4021A, Jiangsu Tianyuan Test Instrument Co., Ltd., Yangzhou, China) was used to measure the impact strength of the PP/SCAB-ECM composite according to GB/T 1043.1-2008 with the 5.5 J capacity at the maximum pendulum height (150°) [40]. The mean value was calculated based on the results of five samples. The universal testing machine (TY-8000A, Jiangsu Tianyuan Test Instrument Co., Ltd., Yangzhou, China) was used to test the flexural strength of the PP/SCAB-ECM composite according to GB/T 9341-2008 [41]. with a speed of 5 mm/min. The mean value was calculated based on the results of five samples.

## 4. Conclusions

In this study, the SCAB-ECM composite nucleating agent was obtained by the physical blending of SCAB and ECM by constructing a hydrogen-bonded self-assembled structure in order to improve the dispersion property of the SCAB in the PP matrix and to exert internal lubrication effects on the PP matrix. The PP/SCAB-ECM composite was prepared by the melting blending method.

The FT-IR results indicate that the stable self-assembled structure was formed by hydrogen bonding interactions between the amide groups of ECM and SCAB. The SEM illustrated that the microscopic morphology of SCAB, which was tightly arranged slender strips, showed a tendency to separate due to the addition of ECM, which demonstrated that the formation of hydrogen-bonded self-assembled structures could improve the dispersion of SCAB in the PP matrix.

The isothermal crystallization kinetic analysis of the PP/SCAB-ECM composite illustrated that the *K* of PP, PP/ECM, PP/SCAB, and PP/SCAB-ECM were 0.0002, 0.0004, 1.1616, and 1.8539, respectively, at the crystallization temperature of 135 °C. The results demonstrated that the addition of ECM played a minor effect on the crystallization properties of PP, and increased the crystallization rate of PP slightly. The crystallization properties of PP/SCAB-ECM were the best, which was attributed to the fact that SCAB formed a hydrogen-bonded self-assembled structure with ECM, which promoted the dispersion of SCAB in the PP matrix. The results of *X_c_* and *t*_1/2_ also verified the conclusion. The isothermal thermodynamic analysis indicated that the ECM formed a hydrogen-bonded self-assembled structure with the SCAB to improve the dispersion of the SCAB. The SCAB-ECM composite nucleating agent reduced the interfacial free energy perpendicular to the molecular chain direction, which facilitated the folding and growth of the molecular chains on the nucleus, thus promoting the improvement of PP crystallization properties. The results of the rotary rheometer indicated that ECM exhibited internal lubrication in the PP, which could effectively reduce the interaction force between the PP molecular chains, improve the flexibility of PP molecular chains, and greatly improve the crystallization ability of the PP. Meanwhile, SCAB-ECM could be better dispersed in the PP matrix due to the formation of hydrogen-bonded self-assembled structures. The addition of the SCAB could greatly improve the impact strength and flexural strength of the PP. Meanwhile, with the formation of the hydrogen-bonded self-assembled structure between the SCAB and ECM, the impact strength of PP/SCAB was further improved by better dispersion in the PP matrix, which allowed a better refinement of the spherical crystal size. The results for POM verified the conclusion that the impact strength varied. But, the ECM played the role of internal lubrication in the PP matrix to reduce the PP intermolecular forces, and when it was deformed by external forces, the molecular chains could slip and rotate with each other, which made the internal friction between the PP molecules decrease, resulting in the decrease in the flexural strength.

## Figures and Tables

**Table 1:** Isothermal crystallization kinetics parameter of the samples.

Sample	*T_c_* (°C)	*T_m_* (°C)	Δ*H*_m_ (J/g)	*X*_c_ (%)	*n*	*K* (min^−n^)	R^2^	*t_1/2_* (min)
PP	130	167.52	60.96	34.44	4.28	0.0189	0.9989	4.86
	133	168.47	61.26	34.61	4.35	0.0016	0.9995	13.76
	135	169.07	46.12	26.06	5.12	0.0002	0.9991	22.21
PP/SCAB	130	165.59	91.76	51.84	3.86	2.9344	0.9921	0.42
	133	168.11	91.85	51.89	3.96	2.0610	0.9918	0.52
	135	169.62	92.65	52.34	3.99	1.1616	0.9979	0.81
PP/ECM	130	165.14	88.04	49.74	4.55	0.0073	0.9999	5.04
	133	166.32	88.68	50.10	4.74	0.0009	0.9999	11.12
	135	166.96	70.82	40.01	5.19	0.0004	0.9999	15.70
PP/SCAB-ECM	130	165.26	92.23	52.09	3.88	3.6939	0.9956	0.24
	133	167.18	93.48	52.81	3.99	2.1759	0.9851	0.40
	135	169.24	92.14	52.05	4.03	1.8539	0.9935	0.56

**Table 2:** Isothermal crystallization thermodynamic parameter of the PP/SCAB-ECM composite.

Sample	*K*_g_ (×10^5^ K^−2^)	Δ*E* (kJ/mol)	σ_e_ (erg/cm^2^)
PP	2.01	209.87	37.72
PP/SCAB	1.25	64.49	23.46
PP/ECM	1.54	119.53	28.90
PP/SCAB-ECM	1.09	51.53	20.46

**Table 3:** Arrhenius equation parameters of the PP/SCAB-ECM composite.

Sample	*lnA* (Pa·s)	*ΔE* (J/mol)	*R^2^*
PP	3.78	34.52	0.99868
PP/SCAB	3.89	44.67	0.99668
PP/ECM	2.60	24.34	0.99176
PP/SCAB-ECM	2.76	24.81	0.99495

**Table 4:** The formula of the PP/SCAB-ECM composite.

Sample	PP (g)	SCAB (g)	ECM (g)
PP	200	-	-
PP/SCAB	200	0.6	-
PP/ECM	200	-	0.6
PP/SCAB-ECM	200	0.6	0.6

## Data Availability

Data are contained within the article.
